# Nervous Interconnection Between the Lesser Occipital and Auriculotemporal Nerves

**DOI:** 10.7759/cureus.25643

**Published:** 2022-06-03

**Authors:** Marc A Gebara, Joe Iwanaga, Aaron S Dumont, R. Shane Tubbs

**Affiliations:** 1 Anatomical Sciences, Tulane University School of Medicine, New Orleans, USA; 2 Neurosurgery, Tulane University School of Medicine, New Orleans, USA

**Keywords:** auriculotemporal nerve, lesser occipital nerve, dermatome, variations, anatomy

## Abstract

The auriculotemporal nerve is one branch of the mandibular portion of the trigeminal nerve, which itself divides into several branches in the temporal and retromandibular regions. The lesser occipital nerve is a cutaneous branch of the cervical plexus and is sometimes implicated in cases of cervicogenic headaches, occipitoparietal headaches, and occipital neuralgia, in general. Here, we present a case of unilateral neural interconnection between the auriculotemporal and lesser occipital nerves thus illustrating the joining of the cervical plexus and trigeminal nerve. A better understanding of the aforementioned nervous anatomy may be valuable for facial reconstructive and nerve transfer procedures, as well as for a variety of other head and neck disorders, e.g., occipital neuralgia.

## Introduction

The auriculotemporal nerve (ATN) arises from the mandibular division of the trigeminal nerve in the infratemporal fossa [1]. The ATN leaves the infratemporal fossa and innervates the parotid gland. In its ascent, the nerve travels closely with the superficial temporal artery and continues superior to the temporomandibular joint to give off communicating branches to the facial nerve [2,3]. Its terminal superficial temporal branches are variable in distribution [4].

The lesser occipital nerve (LON) derives from the ventral rami of the second or third cervical nerve as a cutaneous branch of the cervical plexus [5,6]. The LON ascends along the posterior edge of the sternocleidomastoid muscle (SCM) after curving around its posterior margin and the spinal accessory nerve [6]. This nerve pierces the deep cervical fascia close to the skull base before climbing over the occipital portion, where the auricular branch supplies the upper and medial thirds of the auricular skin and posterior branches innervate the proximal scalp. The LON may interconnect with the greater occipital nerve (GON), great auricular nerves, and occasionally, the facial nerve’s auricular branch [5,6]. The LON usually divides into the terminal medial and lateral branches between the intermastoid line and external occipital protuberance [7].

As variations of the nerves of the head and neck can result in varied clinical presentations their anatomy should be known to clinicians who perform various regional procedures, e.g., anesthetic blockade, and surgeons who operate in these areas. Here, we report an unusual connection between nerves of the temporal and occipital regions.

## Case presentation

**Figure 1:**
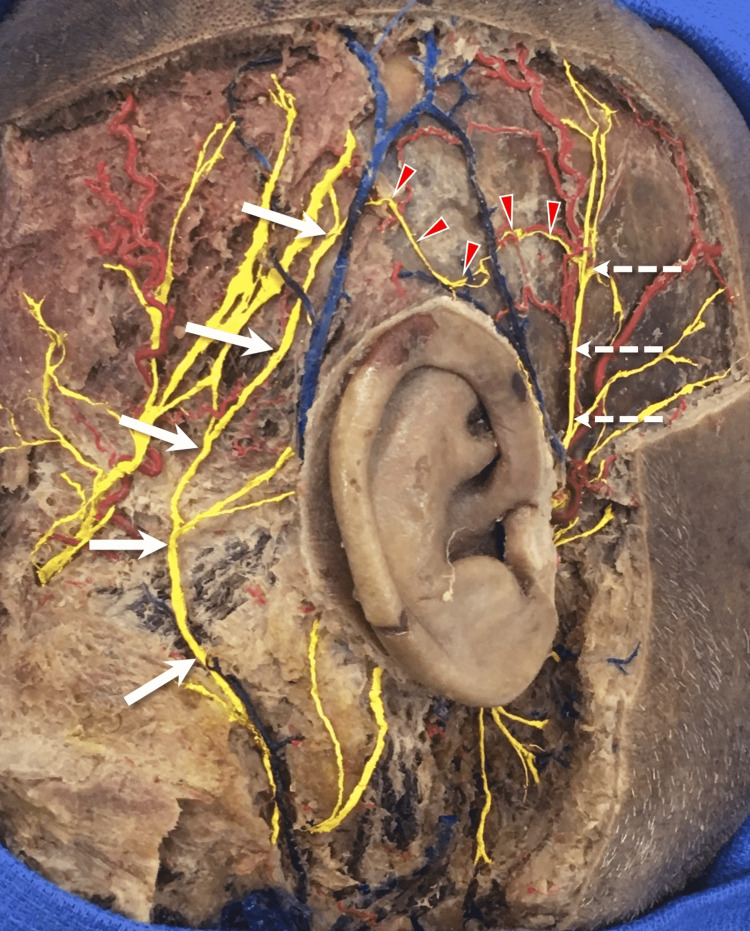
Right-sided cadaveric head showing the auriculotemporal nerve (dotted arrows) and lesser occipital nerve (arrows) with a communicating fiber between the two nerves (arrowheads). Note that all regional nerves are colored yellow, arteries red, and veins blue.

During the routine dissection of a formalin-fixed cadaveric head of a 53-year-old at-death male, an anatomical variation was found in the right temporal region. The superficial temporal branch of the ATN and LON was found to have a communicating branch that united the two nerves superior to the auricle (Figure 1).

The communicating branch (0.4 mm in diameter and approximately 8 cm in length) traveled superior to the apex of the auricle by approximately 3 mm and traveled deep to an arch-shaped vein derived from the superficial temporal vein and a large posterior auricular vein. Traveling from anterior to posteriorly, the communicating branch took on a sinusoidal course. Anteriorly, it joined a posterior division of the superficial temporal branch of the ATN. Posteriorly, it jointed the distal part of the LON at approximately 5 cm superior to the auricle and in line with its posterior border. Additionally, the LON was found to have two intercommunicating branches with the GON and the ATN was seen to send a communication to the temporal branch of the facial nerve. There was no obvious surgical scar or history of trauma in the areas dissected and no other neurovascular variations were noted in these same regions. There was no such intercommunicating nerve branch on the left side. The present study was performed in accordance with the requirements of the Declaration of Helsinki (64th WMA General Assembly, Fortaleza, Brazil, October 2013).

## Discussion

Variations of the ATN

Of the several variations identified, the ATN branching pattern from the trigeminal nerve is the most common [1]. In this case, the most frequently seen are two roots, the superior and inferior roots, but there has been evidence showing up to four roots splitting off the trigeminal nerve [1]. The ATN has been shown to have two branches surrounding the middle meningeal artery when they are identified as pairs [8]. This nerve may even show rare encirclement patterns with the superficial temporal artery [9,10]. The ATN is classically described as having a single main trunk that splits into multiple terminal branches. Iwanaga et al. found that the ATN and its branches show intricate nerve plexuses in the preauricular and temporal areas [4]. They also found neural loops between distal superficial temporal branches and identified two main nerve trunks which have not been previously mentioned in literature. Baumel et al. 1971 asserted that the ATN’s pathway has been often misrepresented in textbooks [11]. They found that in 85 dissections the ATN does not form a loop around the middle meningeal artery. They found the roots formed a long V-shape and this was confirmed by Gülekon and Anil [12]. ATN branch variations in relation to the middle meningeal artery have classically described the upper root lateral to the artery and a lower loop medial to the artery. Gülekon and Anil confirmed this finding as well for most of their cases, but there was one case where the upper root was medial and the lower was lateral to the artery [12]. They found that for ATNs with one root, the number of those with medial were nearly the same as those with roots lateral to the artery [12,13]. Baumel et al., however, found that for single-root situations, the lateral position predominated [11].

Variations of the LON

Duplication and bilateral triplication have been reported for LON. It has also rarely been observed to pass through the posterior cervical triangle’s “carefree region” [14-17]. Furthermore, the LON can sometimes originate from the spinal accessory nerve where C2 connects to it [13]. Ducic et al. noted that the LON and GON travel closer together than many reports in the literature suggest [10]. They also demonstrated that the LON crosses the SCM more superiorly than previously assumed. Lucas et al. identified a situation where a loop from C2 ventral rami encircles the anterior scalene or the levator scapulae [17]. 

The 2000 study by Pantaloni and Sullivan investigated the anatomy of the LON in 19 cadavers as it relates to sensory innervation of the ear to determine the optimal way of avoiding injury during facelift operations [18]. They found that despite the great auricular nerve supplying the majority of sensory innervation to the ear, the LON supplied two-thirds or more of sensation in five of the 19 cadavers. Though the LON was usually seen rising from the posterior edge of the SCM superior to the beginning of the great auricular nerve, the authors found that the LON pierces the subcutaneous plane in varied positions. 

The GON has been known to supply some regions normally supplied by the LON. There have been instances where the LON veers away from its traditional path along the posterior border of the SCM, instead of moving backward into the trapezius muscle’s upper edge before reaching the scalp [19]. The auricular branch of the LON can originate from the GON, usually deriving from the second and third cervical nerves or the loop between them [19]. Communication between the ATN and LON has rarely been reported. Becser et al. 1998 described 2/19 such instances [20]. These authors also described communication between the ATN and GON in 2/20 cases. Our present report on such nervous interconnections may provide physicians with valuable insights into treatment options for a variety of conditions. 

## Conclusions

We present a case of unilateral neural interconnection between the auriculotemporal and LONs thus illustrating the joining of the cervical plexus and trigeminal nerve. Although such communications are normally seen between branches of the cervical plexus and the facial nerve, reports of these interconnections between this plexus and the fifth cranial nerve are scant in the literature. Although the function of such communications is not clear, clinicians might consider these in patients with unusual clinical presentations or during invasive procedures near the ATN or LON.
